# Efficacy and Safety of Shenqu Xiaoshi Oral Liquid Compared With Domperidone Syrup in Children With Functional Dyspepsia

**DOI:** 10.3389/fphar.2022.831912

**Published:** 2022-02-04

**Authors:** Yi Yu, Xiao-Li Xie, Jie Wu, Zhong-Yue Li, Zhi-Gang He, Chun-Jie Liang, Zhong-Qin Jin, Ai-Zhen Wang, Jian Gu, Ying Huang, Hong Mei, Wei Shi, Si-Yuan Hu, Xun Jiang, Juan Du, Chi-Jun Hu, Li Gu, Mao-Lin Jiang, Zhi-Qin Mao, Chun-Di Xu

**Affiliations:** ^1^ Department of Pediatrics, Ruijin Hospital, Shanghai Jiaotong University School of Medicine, Shanghai, China; ^2^ Department of Pediatric Gastroenterology, Chengdu Women’s and Children’s Central Hospital, School of Medicine, University of Electronic Science and Technology of China, Chengdu, China; ^3^ Department of Pediatric Gastroenterology, Shengjing Hospital of China Medical University, Shenyang, China; ^4^ Department of Gastroenterology, Children’s Hospital of Chongqing Medical University, National Clinical Research Center for Child Health and Disorders, Chongqing, China; ^5^ Department of Pediatric Gastroenterology, Children’s Hospital of First People’s Hospital of Chenzhou, Chenzhou, China; ^6^ Department of Pediatrics, Zhanjiang Central Hospital, Guangdong Medical University, Zhanjiang, China; ^7^ Department of Gastroenterology, Children’s Hospital of Soochow University, Suzhou, China; ^8^ Department of Pediatrics, Taizhou Hospital of Traditional Chinese Medicine, Taizhou, China; ^9^ Department of Pediatrics, Taihe Hospital, Shiyan, China; ^10^ Department of Gastroenterology, Children’s Hospital of Fudan University, Shanghai, China; ^11^ Department of Gastroenterology, Wuhan Children’s Hospital, Tongji Medical College, Huazhong University of Science & Technology, Wuhan, China; ^12^ Department of Pediatrics, Tongji Hospital of Tongji University, Shanghai, China; ^13^ Department of Pediatrics, First Teaching Hospital of Tianjin University of Traditional Chinese Medicine, Tianjin, China; ^14^ Department of Pediatrics, The Second Affiliated Hospital of Air Force Military Medical University, Xi’an, China; ^15^ Department of Pediatrics, Jinshan Hospital of Fudan University, Shanghai, China; ^16^ Department of Pediatric Gastroenterology, Maternal and Child Health Hospital of Hubei Province, Tongji Medical College, Huazhong University of Science and Technology, Wuhan, China; ^17^ Department of Pediatrics, Shanghai 10th People’s Hospital, Shanghai, China

**Keywords:** Shenqu Xiaoshi oral liquid, traditional Chinese medicine, functional dyspepsia, domperidone syrup, children

## Abstract

**Background:** Treatment of functional dyspepsia (FD) in children is generally symptomatic and unsatisfactory. Traditional Chinese medicines, such as Shenqu Xiaoshi Oral Liquid (SXOL), have been recommended to alleviate dyspeptic symptoms. However, evidence of their safety and efficacy remains limited to date. AIM: To assess whether 2 weeks of therapy with SXOL was non-inferior to domperidone syrup in children with FD.

**Methods:** In this randomized, double-blind, double-simulated, non-inferiority, multi-center clinical trial, we recruited children (3–14 years) with FD according to the Rome IV criteria from 17 tertiary medical centers across China. Patients were randomly allocated (1:1) to receive SXOL or domperidone syrup for 2 weeks. We compared the participants’ clinical scores from both groups based on the severity and frequency of dyspepsia symptoms according to Rome IV criteria (0, 1, 2, and 4 weeks after randomization). The primary endpoint was the total response rate, which was defined as the proportion of patients with a decrease of 30% or more in the FD symptoms clinical score from baseline, at the end of the 2-weeks treatment. A non-inferiority margin of -10% was set. Secondary endpoints and adverse events were assessed. This trial is registered with www.Chictr.org.cn, number ChiCTR1900022654.

**Results:** Between February 2019 and March 2021, a total of 373 patients were assessed for eligibility, and 356 patients were enrolled and randomized. The clinical response rate at week two was similar for SXOL [118 (83.10%) of 142] and domperidone [128 (81.01%) of 158]; difference 2.09; 95% CI −6.74 to 10.71, thereby establishing non-inferiority. The total FD symptom scores were significantly improved in the two groups at 1-, 2-, and 4-weeks follow-up periods (*p* < 0.005). The decrease in symptom score compared with the baseline were similar between these two groups. Over the total study period, 10 patients experienced at least one treatment-related adverse event [six (3.37%)] in the SXOL group, four [(2.25%) in the domperidone group], although no serious adverse event was noted.

**Conclusion:** Treatment with SXOL effectively improves dyspeptic symptoms and is well tolerated. In addition, it is not inferior to domperidone syrup and leads to sustained improvement in Chinese children with FD.

## Introduction

Functional dyspepsia (FD) presents with chronic symptoms of postprandial fullness, early satiation, or epigastric pain/discomfort when no evidence of systemic, organic, or metabolic disease is found. The Rome IV criteria divide childhood FD into postprandial distress syndrome (PDS) and epigastric pain syndrome (EPS), based on their main symptoms (Hyams et al., 2016). FD is one of the most frequent functional gastrointestinal disorders (FGIDs), affecting approximately 4.5–7.6% of children worldwide (Korterink et al., 2015; Robin et al., 2018). FD in children poses a risk for physical and psychosocial distress, along with school absences and considerable negative financial impact on healthcare (Brook et al., 2010).

Although the exact pathogenesis of FD remains unclear, several pathophysiological mechanisms have been proposed to influence the etiology of FD, such as gastroduodenal motor dysfunction, visceral hypersensitivity, low-grade gastroduodenal inflammation, *Helicobacter pylori* infection, mental stress, and/or abnormal central nervous system processing through the gut-brain axis (Koppen et al., 2017; Walker et al., 2019; Shava et al., 2021). Most pediatricians commonly personalize the patient’s medical treatment, including dietary modifications, prokinetics, antacids, antispasmodics, antidepressants, and analgesics according to their predominant symptoms and associated comorbidities (Hyams et al., 2016; Browne et al., 2018; Masuy et al., 2019). Nonetheless, their effectiveness remains unsatisfactory. In addition, the potential side effects of long-term use of these synthetic drugs cause concerns (Perez and Youssef, 2007; Yang et al., 2017). Therefore, the prevalence of complementary and alternative medicine, including traditional Chinese medicine (TCM), applied to FGIDs continues to increase worldwide (Deutsch et al., 2020). Among these options, the role of TCM is difficult to ignore in the treatment of FD in children (Saad and Chey, 2006; Yeh and Golianu, 2014).

According to TCM theory, the lesion producing dyspepsia is located in the stomach and involves the liver and spleen. Spleen deficiency and qi stagnation are considered the core pathological mechanism (Lv et al., 2017). Thus, a variety of TCM-based therapies have been developed for treating FD, such as oral Xiangsha Liujunzi granules (Saad and Chey, 2006) and acupuncture treatment (Sun et al., 2018), whose effectiveness and safety have been validated. Shenqu Xiaoshi Oral Liquid (SXOL) is a newly developed, patented Chinese traditional medicine. The formulation is based on the ancient compounds included in Baohe Pills, Sijunzi Decoction, and Shaoyao Gancao Decoction, which promote stomach digestion, ameliorate spleen dysfunction in transportation, regulate qi, and alleviate epigastric pain. However, evidence regarding the clinical efficacy and safety of SXOL is yet limited.

The dopamine antagonist domperidone appears to have maximal prokinetic effect in the upper gastrointestinal (GI) tract and is effective for FD symptoms such as early satiety, bloating, anorexia, nausea, vomiting, epigastric pain, and regurgitation in both adults and children (Barone, 1999; Yang et al., 2017; Karunanayake et al., 2018; Pittayanon et al., 2019). Additionally, short-term usage of domperidone did not increase adverse events compared with patients that received a placebo (Karunanayake et al., 2018; Pittayanon et al., 2019). A similar randomized controlled trial (RCT) investigated the influence of hippophae rhamnoides on appetite factors and gastric emptying in children with FD, using domperidone as positive control (Xiao et al., 2013).

Although SXOL has already been clinically applied for the treatment of FD in pediatric patients, there is still a lack of solid evidence regarding its clinical efficacy and safety from well-designed, multicenter, double-blind, randomized, and controlled clinical trials. In the present study, we aimed to conduct a positive-controlled trial using one of the available traditional treatment options to support the use of SXOL in FD children. Hence, this multicenter RCT assessed whether 2 weeks of therapy with SXOL was non-inferior to domperidone syrup in terms of the response rate observed in children with FD.

## Materials and methods

### Subjects

Subjects were recruited by pediatric gastroenterologists in the outpatient clinics in 17 tertiary medical centers across China between February 2019 and March 2021. Children and adolescents were considered eligible subjects if they met the following inclusion criteria: 1) Outpatients aged 3–14 years old. 2) Diagnosed with FD, according to Rome IV criteria (Hyams et al., 2016). 3) Informed consent was obtained from the parents/guardians of the participant. If the child was more than 10 years old, additional informed consent would be required from the participant. 4) During the 2-weeks lead-in period, no relevant drugs for the treatment of dyspepsia were used, good eating habits have been established, and the FD symptoms still existed. In addition, subjects would be excluded from the study if they met any of the following exclusion criteria: 1) Dyspepsia caused by organic diseases, such as acute and chronic gastroenteritis, peptic ulcer, history of gastrointestinal surgery, acute and chronic hepatitis, anorexia nervosa, and anorexia caused by certain drugs; 2) Simultaneous treatment that can cause constipation or enhanced GI motility; (3) Moderate or severe malnutrition; 4) Serious primary diseases of the cardiovascular, nervous, respiratory, hepatobiliary and urinary systems; 5) Mental disorders, intellectual disabilities, and/or communication impairments; 6) Allergy to the ingredients of SXOL or domperidone syrup; 7) Participated in a clinical trial within the past 12 weeks; 8) Individuals deemed unsuitable for this clinical trial.

This RCT was approved by the Medical Research Ethics Committee of Ruijin Hospital Affiliated to Shanghai Jiaotong University School of Medicine and by the independent ethics committee of each center. The study was conducted in accordance with the principles of the Declaration of Helsinki. Informed consent was obtained from parents/guardians of each participant under 10 years old. If the participant was over 10 years old, informed consent was given by the parents/guardians and the participant.

### Trial Design

A randomized, double-blinded, double-analog, positive-controlled study design was used in accordance with the SPIRIT 2013 explanation and elaboration: guidance for protocols of clinical trials (Chan et al., 2013). The study was divided into three stages: a 2-weeks screening period, a 2-weeks treatment period, and a 2-weeks taper period. During the 2-weeks screening period, patients received education regarding behavioral modifications of diet through family training. Then, participants were randomized into the SXOL or domperidone group in a 1:1 ratio using the block randomization method. During the trial, no additional TCM or western drugs related to the treatment of FD were allowed. Patients were evaluated at 0, 1, 2, and 4 weeks after randomization. This trial was registered at chictr.org.cn (no. ChiCTR1900022654). All authors had access to the study data as well as reviewed and approved the final manuscript.

### Interventions

The SXOL group received SXOL plus placebo-1, while the domperidone group received domperidone syrup plus placebo-2. SXOL was provided by Jiangsu Pharmaceutical Co., Ltd., Yangzijiang Pharmaceutical Group; national medicine permission number Z20153035. SXOL should be taken half an hour after meals, three times a day. The dosage was determined by age according to the formal package inserts of SXOL: 3–4 years old, 5 ml/dose; 5–14 years old 10 ml/dose. Domperidone syrup, manufactured by Xian Janssen Pharmaceutical Co., Ltd., should be taken 15 min before meals, three times a day. The dosage was determined by age according to the formal package inserts of domperidone syrup: 3 years old, 4ml/dose; 4–6 years old 5 ml/dose; 7–9 years old 7 ml/dose, 10–12 years old 9 ml/dose, 13–14 years old 10 ml/dose. The placebo −1 and −2, provided by Jiangsu Pharmaceutical Co., Ltd., Yangzijiang Pharmaceutical Group, did not have any drug component and were each similar to domperidone syrup and SXOL, respectively, in terms of shape, texture, color, odor, taste, adjuvants, and dosage. Subjects, investigators, research coordinators, and statisticians were blinded to treatment assignment until analyses were completed. Patients returned any remaining study drug and empty bottles at each visit (compliance defined as ingestion of the intended dose over 80% and under 120%).

### Assessments

During this trial, we assessed semi-quantitative scores on FD symptoms, adverse events (AEs), clinical laboratory parameters, vital signs, and physical exams. The assessments schedule is shown in Supplementary Table S1. We focused on the severity and/or frequency of eight major symptoms, namely postprandial fullness, early satiation, epigastric pain or burning, loss of appetite, decreased food intake, nausea or vomiting, belching, and defecation frequency, totally 11 scoring items. Each item scored 0–3 points according to its degree: thus, the highest total score was 33 points. Higher scores in a patient represented more severe symptoms. The semi-quantitative symptom scoring standard is shown in Supplementary Table S2. The patient-reported symptom scores were evaluated at day 0, week 1, week 2, and week 4. Efficacy index (EI) was calculated as (points before treatment—points after treatment)/points before treatment ×100%. Criteria for curative effect comparing to the baseline were as follows: ① symptom disappearance: the main symptoms disappeared or almost disappeared, EI ≥ 95%; ② significant response: the main symptoms were significantly improved, 70% ≤ EI<95%; ③ response: the main symptoms were improved to a meaningful degree, 30% ≤ EI <70%; ④ invalid: the main symptoms were slightly improved, or even aggravated, EI <30%. The symptom disappearance rate was calculated as the proportion of subjects whose clinical symptoms disappeared. The same approach was used for the rest of the categories, obtaining a significant response, response, and invalid rates. We also assessed the improvement of body weight and diet time. Additionally, the treatment emergent adverse events (TEAEs) and treatment related adverse events (TRAEs) determined by researchers were recorded as safety data.

### Endpoints

The primary endpoint was the response rate 2 weeks after randomization, which was defined as the proportion of patients whose composite FD symptom score decreased more than 30% compared with baseline. The secondary endpoints included: the clinical symptom disappearance and significant response rates 2 weeks after randomization; the clinical symptom disappearance, significant response and response rates 1 and 4 weeks after randomization; the total symptom score reductions relative to the baseline, each single symptom score reduction relative to the baseline, and the improvement of body weight and diet time at each follow-up time point. The safety endpoints included the incidence of TEAEs, TRAEs, and serious adverse events, and the percentage of subjects who experienced adverse events.

### Sample Size and Statistical Analysis

The sample size was determined by assuming that the total response rate of both the domperidone syrup and Shenqu Xiaoshi Oral Liquid was nearly 90% according to the results of a previous phase III clinical trial (Deng et al., 2021). A one-sided normal approximation test for non-inferiority with a margin of −10% and *α* = 0.05 showed that 142 patients per treatment group would provide a power of 80%. To adjust for up to a 20% dropout rate, the sample size was set at 178 patients per treatment group.

All statistical analyses were performed using SAS software (version 9.4), and tests were done at a 0.05 significance level unless otherwise noted. The patients who took medication at least once after randomization and have corresponding post-medication evaluations were analyzed as the full analysis set (FAS). Missing data about total symptom score at week two in FAS were imputed by the last-observation-carried-forward method. Patients in FAS who have good treatment compliance (actual dosage/expected dosage (daily dosage*expected days) = 80–120%) and have complete the case report form were analyzed as per protocol set (PPS). Safety set (SS) includes all patients who received study treatment at least once with safety assessment during the trial. The primary endpoint was analyzed in the FAS and PPS, and secondary endpoints were analyzed in the FAS. The safety of the treatment was analyzed in the SS.

Continuous data were presented as the mean ± standard deviation (SD) and analyzed using student’s t-test, while categorical data were presented as numbers (percentages) and analyzed using the chi-square test or Fisher exact test. For the primary endpoint, the total effective rate response rate and its 95% confidence interval (CI) for each group were calculated using the Clopper-Pearson method. The rate difference between the two groups and their 95% CI were calculated using the Newcombe-Wilson method. Non-inferiority was declared if the lower limit of the CI was −10%. For the secondary endpoint expressed as continuous variables (total symptom score, individual symptom score, weight, amount of food intake, and diet time), ANCOVA models were used to compare the difference of change from baseline between two groups and calculate its 95% CI. Total response rates were also compared between the SXOL and domperidone groups in certain subgroups, such as age (≤6 years old; >6 years old), sex (boy; girl), body mass index (BMI) (≤15; >15), type of FD (EPS; PDS; EPS and PDS), education level of parents (below college; college or above), course of disease (≤3 months; >3 months), among others.

## Results

### Patient Baseline Characteristics

**FIGURE 1:**
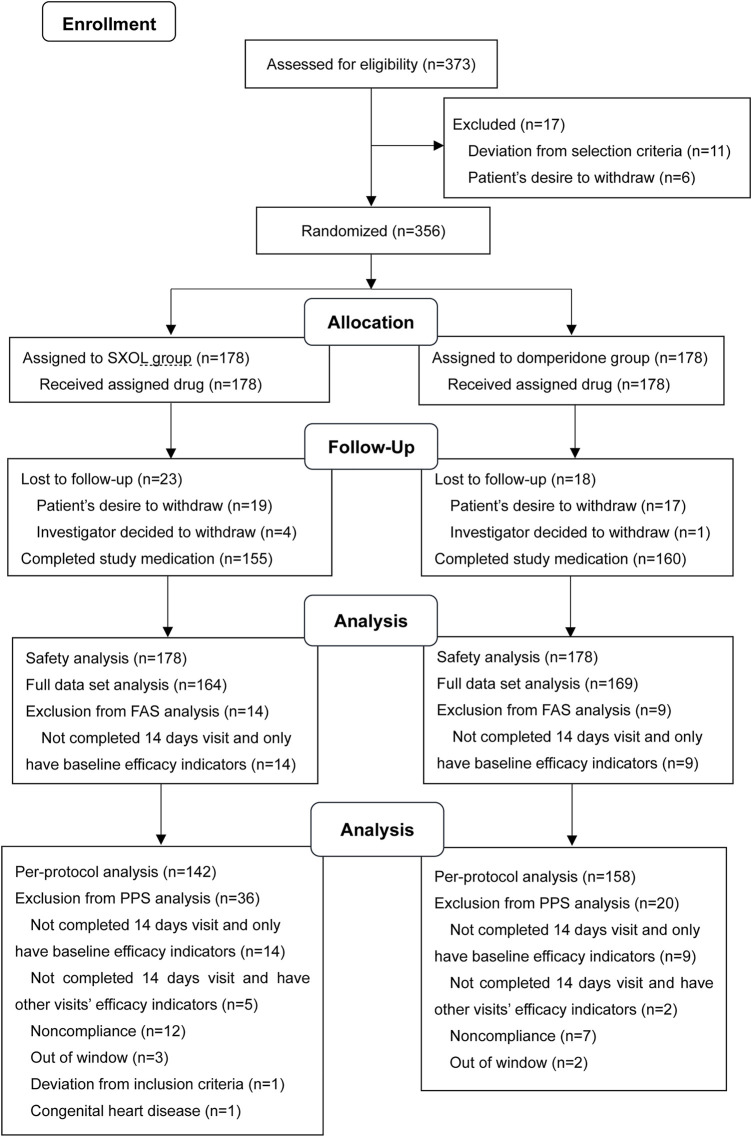
Flow diagram.

In this study, 373 outpatients with a suspected diagnosis of FD were screened, of whom 17 were excluded. Consequently, a total of 356 patients were enrolled and randomly assigned to the SXOL group (*n* = 178) or domperidone group (*n* = 178) at week 0. Ultimately, 23 failed to enter the FAS, whereas 56 failed to enter the PPS. Patient enrolment, randomization, and follow-up are shown in Figure 1. The drop-out rate did not significantly differ between these two groups. Overall, patient demographics and disease characteristics (age, gender, BMI, subtypes proportion, total clinical scores, etc.) at baseline showed no significant differences between the groups in FAS analysis (all *p* > 0.05), thus suggesting the backgrounds were comparable between these groups before treatment (Table 1).

**TABLE 1:** Baseline characteristics of the study population in FAS.

	Total (*n* = 333)	SXOL group (*n* = 164)	Domperidone group (*n* = 169)	*p* Value
Age (years old), mean ± SD	6.78 ± 2.54	6.63 ± 2.52	6.92 ± 2.56	0.290
Female, *n* (%)	132 (39.64%)	62 (37.80%)	70 (41.42%)	0.500
BMI (kg/m^2^), mean ± SD	15.22 ± 2.09	15.29 ± 2.39	15.16 ± 1.76	0.595
Rome Ⅳ diagnosis of FD	—	—	—	0.472
PDS	122 (36.64%)	64 (39.02%)	58 (34.32%)	—
EPS	52 (15.62%)	22 (13.41%)	30 (17.75%)	
PDS and EPS	159 (47.75%)	78 (47.56%)	81 (47.93%)	
Disease duration (weeks), mean ± SD	43.92 ± 51.59	45.66 ± 58.00	42.24 ± 44.61	0.548
Drug use 3 months before visit, N (%)	87 (26.13%)	39 (23.78%)	48 (28.40%)	0.337
Symptom total score, mean ± SD	9.51 ± 3.94	9.74 ± 3.96	9.29 ± 3.91	0.300
Individual symptom score, mean ± SD	—	—	—	—
Postprandial fullness—severity	0.71 ± 0.71	0.74 ± 0.71	0.69 ± 0.72	0.461
Postprandial fullness—frequency	0.87 ± 0.95	0.95 ± 0.99	0.80 ± 0.91	0.144
Early satiation—severity	1.19 ± 0.91	1.21 ± 0.90	1.17 ± 0.93	0.677
Early satiation—frequency	1.24 ± 0.95	1.29 ± 0.96	1.19 ± 0.94	0.349
Epigastric pain—severity	0.91 ± 0.79	0.90 ± 0.81	0.91 ± 0.78	0.919
Epigastric pain—frequency	1.14 ± 1.00	1.13 ± 1.01	1.15 ± 0.99	0.814
Loss of appetite—severity	1.00 ± 0.67	1.05 ± 0.63	0.95 ± 0.70	0.189
Reduced food intake—severity	1.20 ± 0.85	1.21 ± 0.83	1.18 ± 0.88	0.798
Nausea or vomiting—severity	0.41 ± 0.62	0.44 ± 0.67	0.37 ± 0.57	0.331
Belching—frequency	0.38 ± 0.64	0.35 ± 0.60	0.40 ± 0.67	0.539
Defecation—frequency	0.47 ± 0.55	0.47 ± 0.55	0.48 ± 0.56	0.872

PDS: postprandial distress syndrome; EPS: epigastric pain syndrome.

### Primary Endpoint

Based on FAS analysis, 136 (82.93%, 95% CI 76.28–88.35) of 164 patients assigned to SXOL group and 137 (81.07%, 95% CI 74.33–86.67) of 169 patients assigned to domperidone group achieved a clinical response at week 2 [difference 1.86% (95% CI −6.45–10.11)]. Similar results were observed for the PPS at week 2, with 118 (83.10%, 95% CI 75.90–88.86) of 142 in the SXOL group and 128 (81.01%, 95% CI 74.02–86.81) of 158 in the domperidone group achieving response [difference 2.09% (95% CI −6.74–10.71)] (Table 2). Overall, prespecified non-inferiority criteria were met for both populations and SXOL was considered non-inferior to domperidone syrup.

**TABLE 2:** Response, significant response and symptoms disappear rate at weeks 1, 2, and 4.

	SXOL group		Domperidone group		Difference	*p* Value
	*n*	*n* (%, 95% CI)	*n*	*n* (%, 95% CI)	(%; 95% CI)	
Primary outcome	—	—	—	—	—	—
Response rate (Week 2)	—	—	—	—	—	—
FAS	164	136 (82.93, 76.28–88.35)	169	137 (81.07, 74.33–86.67)	1.86; −6.45–10.11^NI^	—
PPS	142	118 (83.10, 75.90–88.86)	158	128 (81.01, 74.02–86.81)	2.09; −6.74–10.71 ^NI^	—
Secondary outcomes	—	—	—	—	—	—
Week 1	—	—	—	—	—	—
Response rate	164	102 (62.20, 54.30–69.64)	169	106 (62.72, 54.96–70.03)	−0.53; −10.83–9.77	0.921
Significant response rate	164	27 (16.46, 11.14–23.04)	169	41 (24.26, 18.01–31.44)	−7.80; −16.33–0.89	0.078
Symptoms disappear rate	164	7 (4.27%, 1.73–8.60)	169	10 (5.92%, 2.87–10.61)	−1.65; −6.77–3.40	0.494
Week 2	—	—	—	—	—	—
Significant response rate	164	75 (45.73, 37.94–53.68)	169	77 (45.56, 37.90–53.39)	0.17; −10.41–10.75	0.975
Symptoms disappear rate	164	24 (14.63, 9.61–20.99)	169	29 (17.16, 11.80–23.71)	−2.53; −10.40–5.41	0.529
Week 4	—	—	—	—	—	—
Response rate	164	140 (85.37, 79.01–90.39)	169	148 (87.57, 81.63–92.14)	−2.21; −9.69–5.21	0.556
Significant response rate	164	92 (56.10, 48.15–63.82)	169	99 (58.58, 50.76–66.09)	−2.48; −12.96–8.06	0.647
Symptoms disappear rate	164	52 (31.71, 24.67–39.42)	169	52 (30.77, 23.91–38.32)	0.94; −8.94–10.82	0.853

NI: Non-inferior.

### Secondary Endpoints

After 2 weeks of treatment, FAS analysis revealed that 45.73% (95% CI 37.94–53.68) of patients from the SXOL group achieved significant response in comparison to 45.56% (95% CI 37.90–53.39) of patients from the domperidone group, with a non-statistical difference (*p* = 0.975). In addition, 14.63% (95% CI 9.61–20.99) of patients from the SXOL group achieved symptom disappearance in comparison to 17.16% (95% CI 11.80–23.71) in the domperidone group according to FAS analysis, without a statistical difference (*p* = 0.529). The total response rate at week 4 (2 weeks after withdrawal) was well maintained and similar between the groups (85.37 vs. 87.57%, *p* = 0.556). Similarly, the significant response rate and symptom disappearance rate were well maintained at week 4 (Table 2).

**FIGURE 2:**
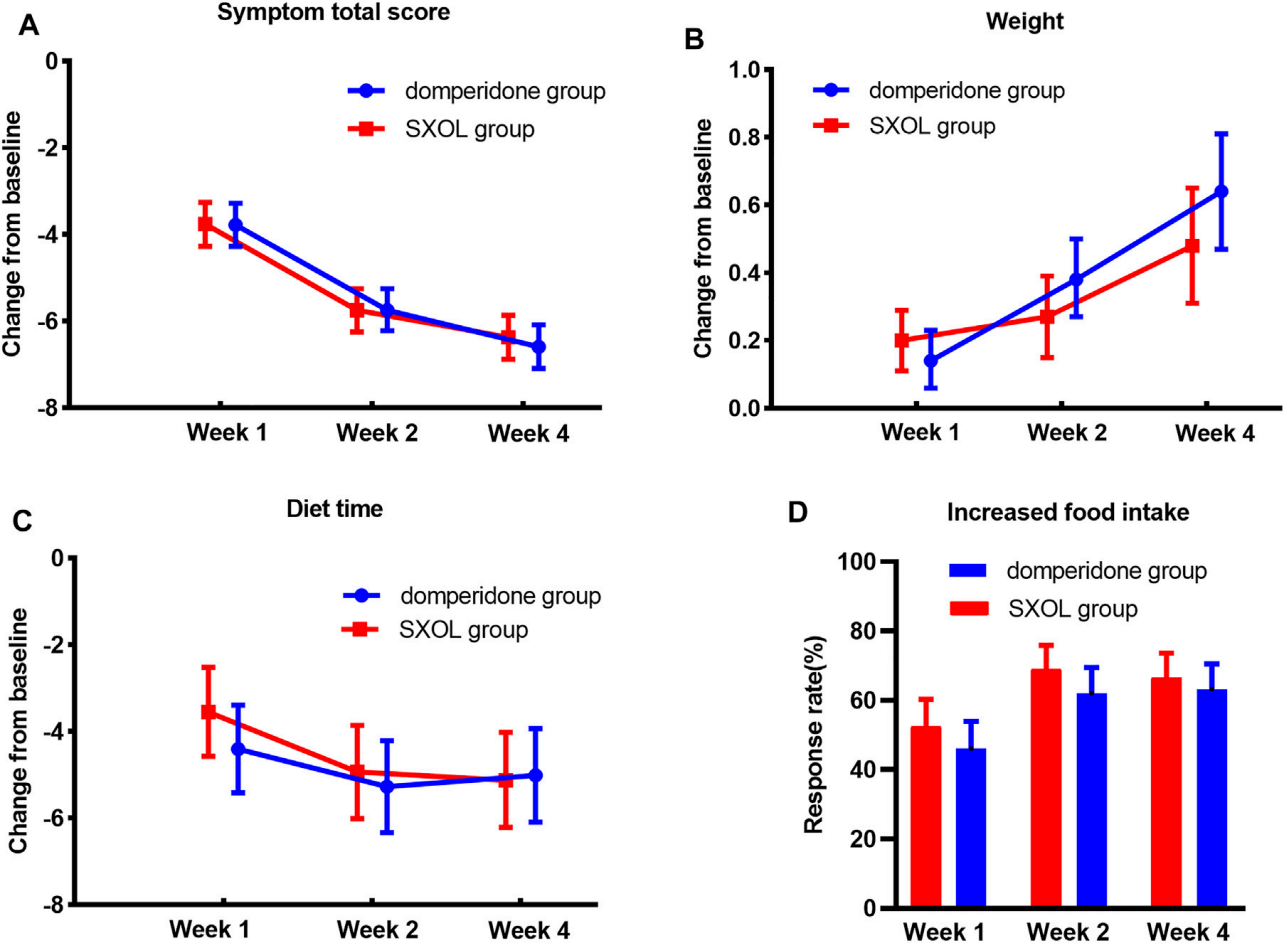
Treatment effects in different time point. **(A)** Decrease of symptom total score compare with baseline. **(B)** Weight gaining from baseline (kg). **(C)** Shortening of diet time from baseline (minute). **(D)** Response rate of food intake increasing more than 30%.

The total scores of clinical symptoms were significantly reduced relative to the baseline at weeks 1, 2, and 4 after randomization in both groups. Furthermore, there was no significant difference in the degree of improvement between the two groups (*p* > 0.05), as illustrated in Figure 2A. Moreover, the two groups achieved good results in gaining weight, shortening eating time, and increasing food intake, but there was no statistical difference between them (Figure 2).

**FIGURE 3:**
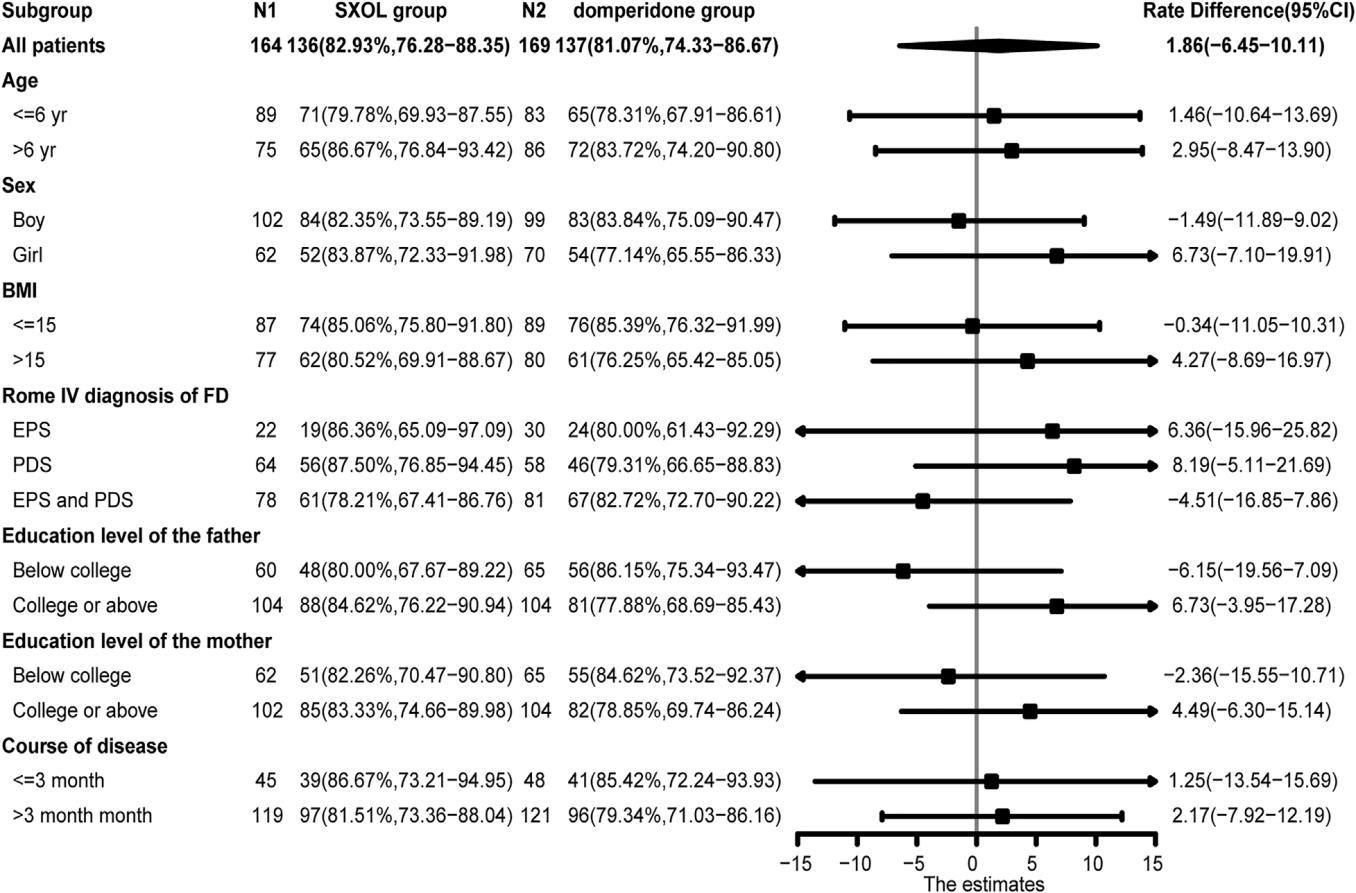
Forest plot (Response rate at week two in different subgroups).

There was no significant difference in the response rate at week two between the two groups when comparing the different subgroups based on age, sex, BMI, subtype classification, education level of parents, and course of disease (*p* > 0.05) (Figure 3). Concerning the evaluation of the individual upper GI symptoms, only early satiety showed a significantly less improvement at week two in the SXOL group [95% CI −1.37 (−1.56–1.17)] compared with the domperidone group [95% CI −1.69 (−1.88–1.50)]. Although domperidone seemed more effective than SXOL in relieving early satiety symptoms [95% CI 0.32 (0.05–0.59), *p* = 0.02], SXOL [95% CI −0.61 (−0.69–0.52)] had a potential advantage over domperidone [95% CI −0.50 (−0.58–0.42)] in the improvement of epigastric pain severity at 2 weeks after the intervention; however, no statistical difference was found between groups [95% CI −0.11 (−0.23–0.01), *p* = 0.08], as shown in the Supplementary Table S3.

### Adverse Events

A total of 60 adverse events occurred in 41 patients during the trial. Twenty-six patients (14.61%) in the SXOL group experienced 29 TEAEs, of which six (3.37%) patients experienced TRAEs and no serious adverse event was noted. In the domperidone group, 25 patients (14.04%) experienced 31 TEAEs, of which four (2.25%) patients experienced TRAEs and no serious adverse event was noted. This information is shown in Table 3.

**TABLE 3:** Safety analysis.

	SXOL group (*N* = 178)		Domperidone group (*N* = 178)	
	Time	*n* (%)	Time	*n* (%)
TEAE^a^	29	26 (14.61%)	31	25 (14.04%)
TRAE^b^	6	6 (3.37%)	6	4 (2.25%)
Vomit	1	1 (0.56%)	1	1 (0.56%)
Stomach ache	2	1 (0.56%)	0	0 (0%)
Creatinine increasing	0	0 (0%)	1	1 (0.56%)
Hematuria	1	1 (0.56%)	0	0 (0%)
High urine white blood cells	1	1 (0.56%)	0	0 (0%)
Early breast Development	0	0 (0%)	1	1 (0.56%)
Atopic dermatitis	0	0 (0%)	1	1 (0.56%)
Headache	0	0 (0%)	1	1 (0.56%)
Gastroenteritis	0	0 (0%)	1	1 (0.56%)
Abnormal liver function	1	1 (0.56%)	0	0 (0%)
Serious TEAE	0	0	0	0
Serious TRAE	0	0	0	0
TEAE caused interruption of treatment	2	2 (1.12%)	1	1 (0.56%)
TEAE caused withdraw	3	3 (1.69%)	1	1 (0.56%)
TEAE caused death	0	0	0	0

a TEAE: treatment emergent adverse events.

b TRAE: treatment related adverse events.

## Discussion

FD is a highly disabling FGIDs for children, as it is associated with negative health outcomes and decreased psychosocial functioning, leading to frequent treatment-seeking behaviors and huge demand for medical resources (Perez and Youssef, 2007; Brook et al., 2010; Mani et al., 2020). As the exact pathogenesis of FD is unknown, treatment of FD in pediatric patients is challenging, mostly symptomatic, not well defined, and often used for months or years (Hyams et al., 2016; Browne et al., 2018). This RCT confirmed that treatment with SXOL for 2 weeks effectively alleviated FD symptoms with reasonable toleration, which was not inferior to domperidone syrup, and sustained improvement after withdrawal in Chinese children.

A considerable number of herbal products have been recommended alone or in combination for the treatment of dyspeptic symptoms (Thompson Coon and Ernst, 2002; Yang et al., 2013; Zhang et al., 2013; Giacosa et al., 2015; Fifi et al., 2018). For example, Zhizhu Kuanzhong is superior to placebo in the treatment of PDS due to its higher response rate (54.7 vs 38.8%) (Xiao et al., 2019). A recent meta-analysis has demonstrated that Rikkunshito, which is a Japanese Kampo-herbal medicine, exhibited a significantly higher total clinical efficacy rate compared with western medicine (relative risk = 1.21, 95% CI 1.17 to 1.25, *p* < 0.001) (Ko et al., 2021).

SXOL is composed of the following TCM crude drugs: Liu Shenqu (Massa Medicata Fermentata), charred hawthorn (Crataegi Fructus), charred malt (Hordei Fructus Germinatus), Paeoniae Radix Alba, Codonopsisradix, Poria, Rhizoma Atractylodis Macrocephalae Stir-fried with Bran, Aucklandiae Radix, Fructus Amomi, Rhizoma Corydalis, Glycyrrhizae Radix Et Rhizoma Praepapata Cum Melle. The formulation acts by promoting stomach digestion, invigorating the spleen, regulating qi, and alleviating epigastric pain according to TCM theory. It can be used for children with weak spleen and stomach caused by improper feeding or unregulated diet, as well as for anorexia, loss of appetite, and reduced appetite caused by the diet stagnation syndrome. The indications of SXOL in this trial were similar to those published, which recommended this concoction for children with FD. The sovereign drugs of SXOL, including Liu Shenqu, charred hawthorn, and charred malt, which are commonly known as “Jiao Sanxian”. Their effects include invigorating the spleen and appetizing the stomach, eliminating accumulation, and resolving stagnation, by respectively promoting the digestion of pasta, meat, and rice. For these reasons, “Jiao Sanxian” is a widely used medicine for enhancing digestion and improving stagnation in China. Recently, a basic study reported that “Jiao Sanxian” can improve functional dyspepsia in rats and the mechanism involves regulating the secretion of brain-gut peptides, improving the disorder of intestinal flora and ameliorating multi-metabolic pathways (Liu et al., 2021).

Other main components, such as Hawthorn, Atractylodis Rhizoma, Codonopsis, Amomi Fructus, and Rhizoma Corydalis, have been used as traditional medicines and/or food materials, which have extensive pharmacological activities in gastrointestinal function regulation and almost no toxicity. Hawthorn is a commonly used TCM for treating dyspepsia, which has been proven to improve gastrointestinal motility by plasma metabolomics, particularly in relation to amino acid metabolism and primary bile acid biosynthesis (Wang et al., 2021). A recent review suggested that Atractylodis Rhizoma improved gastrointestinal function by stimulating gastric emptying, small intestinal motility, or anti-gastrointestinal mucosal injury (Zhang et al., 2021). Codonopsis regulates gastrointestinal function, neuroprotection, endocrine function, etc., (Gao et al., 2018). Evidence on Amomi Fructus has confirmed that it exerts gastrointestinal protection, along with exhibiting anti-inflammatory, analgesic, antidiarrheal, and anti-microbial activities (Suo et al., 2018). Based on prior research, Rhizoma Corydalis has analgesic, antidepressive, anti-anxiety, acetylcholinesterase inhibitory, anti-gastrointestinal ulcer, and anti-inflammatory effects (Tian et al., 2020).

The present study was the first multicenter, positive-controlled RCT to investigate the efficacy of SXOL in alleviating symptoms of FD in children and adolescents, according to the Rome IV criteria, with the purpose of providing an additional effective prescription for the clinical treatment of this disease. In this RCT, non-inferiority was established when the SXOL group was compared with the domperidone group in the FAS and PPS analysis. This was demonstrated by the 95% CI for the treatment difference in the primary endpoint (total response rate at week 2) falling within the prespecified margins, which means that SXOL was as useful as domperidone in children with FD. Furthermore, we found benefits from both the use of domperidone as a prokinetic agent and of SXOL as a TCM in the treatment of FD in children. This study indicated that the total clinical scores of dyspeptic symptoms were significantly reduced relative to the baseline at 1, 2, and 4 weeks after randomization in both SXOL and domperidone syrup groups (*p* < 0.05), and the degree of reduction was similar between the two groups (*p* > 0.05). Symptoms did not rebound after drug withdrawal, which was an important efficacy indicator that should be evaluated for FD treatment. The beneficial effects in the total clinical scores and the total response rate were maintained for at least 2 weeks after withdrawal treatment in both groups.

The gastrointestinal motor disorder is considered a major pathophysiological mechanism underlying symptoms of FD, which leads to impaired myoelectric activity, gastric accommodation, and gastric emptying (Sanger and Alpers, 2008). Domperidone is a prokinetic agent which markedly alleviates dyspeptic symptoms; however, the potential adverse reactions (especially the cardiac and neural toxicity) limit its long-term clinical application (Reddymasu et al., 2007; Yang et al., 2017; Vijayvargiya et al., 2019; Sayuk and Gyawali, 2020). According to this RCT, SXOL may be a reasonable alternative to conventional prokinetic drugs to improve the symptoms of FD children. Moreover, according to the theory of TCM and relevant basic research, SXOL may also have several additional effects besides the prokinetic effect, which is helpful to alleviate epigastric pain and promote digestion.

Although there were no meaningful differences in the response rate at week two among the different subgroups based on the forest plot analysis (*p* > 0.05), SXOL might potentially be better for girls suffering from FD [95% CI 6.73 (−7.10–19.91)]. In addition, considering different subtypes, children diagnosed with either PDS [95% CI 8.19 (−5.11–21.69)] or EPS [95% CI 6.36 (−15.96–25.82)] might respond better to SXOL, but domperidone might be more effective for those overlapping PDS and EPS [95%CI −4.51 (−16.85–7.86)]. SXOL might be more effective for children whose fathers have college or above education level [95%CI 6.73 (−3.95–17.28)] (Figure 3). In terms of the improvement of single symptoms, SXOL seemed to have advantages in alleviating upper abdominal pain, nausea or vomiting, and belching, while domperidone syrup seems to have a better effect on early satiation and loss of appetite. Although there were no statistically significant differences between groups in these single symptoms, increasing the sample size or prolonging the course of treatment may further clarify the efficacy target of the two therapeutic drugs on FD.

Treatments were well tolerated throughout our study. Similar proportions of patients experienced at least one treatment-related adverse event between the test and control group (3.37 vs. 2.25%). In addition, rates of discontinuation due to treatment-related adverse events were similar between groups: therefore, SXOL was considered to be well tolerated. Studies have shown that domperidone was effective for the symptomatic relief of FD. However, considering the adverse events related to domperidone, such as intestinal colic, hyperprolactinemia, diarrhea, headache, and skin scare, solely a short-term use of this drug would be recommended (Yang et al., 2017). TCM can be used as another safe treatment option instead of chemical synthetic medicines.

Our study included children from 17 centers throughout China to ensure the extrapolation of the results through reduction of response bias to the intervention. Additionally, the subjects, parents, investigators, and statistician were blinded for the received treatments, which eliminated subjective bias and personal preferences that might appear in the consciousness of experimenters and participants. Another strength of the study was the follow-up after withdrawal, which allowed us to observe continuous improvement in symptoms.

This study also had several limitations. First, it was not powered to show statistically non-inferiority between SXOL and domperidone syrup for the marked response rate and the significant advantage for EPS or PDS between groups, which might be due to insufficient sample size. The second limitation was that the duration of treatment and follow-up after withdrawal might not be sufficient for efficacy and safety observations. Third, we did not screen and analyze according to the dialectical theory of traditional Chinese medicine, which might be more helpful to find the most suitable indication population of SXOL. Further studies that control for the above-mentioned limitations are needed to confirm the results of this study. Finally, SXOL is composed of 11 TCM ingredients and its complex action mechanisms warrant further investigation.

## Conclusion

In conclusion, despite the high prevalence of FD in children, gold standards are not available for its treatment. This RCT is, to our knowledge, the first investigation of the therapeutic efficacy of a TCM (Shenqu Xiaoshi Oral Liquid) powered to show non-inferiority to domperidone syrup in children with FD. Additionally, it shows scientific and confirmatory evidence of extrapolation of SXOL. The results demonstrate that SXOL was well tolerated and that it does not present clinically meaningful differences in compliance and safety compared with domperidone syrup. Therefore, SXOL can be considered a novel efficacious treatment strategy to help children with FD.

## Data Availability

The original contributions presented in the study are included in the article/Supplementary Material, further inquiries can be directed to the corresponding author.
