# Corrigendum: Efficacy and Safety of Shenqu Xiaoshi Oral Liquid Compared With Domperidone Syrup in Children With Functional Dyspepsia

**DOI:** 10.3389/fphar.2022.920925

**Published:** 2022-06-13

**Authors:** Yi Yu, Xiao-Li Xie, Jie Wu, Zhong-Yue Li, Zhi-Gang He, Chun-Jie Liang, Zhong-Qin Jin, Ai-Zhen Wang, Jian Gu, Ying Huang, Hong Mei, Wei Shi, Si-Yuan Hu, Xun Jiang, Juan Du, Chi-Jun Hu, Li Gu, Mao-Lin Jiang, Zhi-Qin Mao, Chun-Di Xu

**Affiliations:** ^1^ Department of Pediatrics, Ruijin Hospital, Shanghai Jiaotong University School of Medicine, Shanghai, China; ^2^ Department of Pediatric Gastroenterology, Chengdu Women’s and Children’s Central Hospital, School of Medicine, University of Electronic Science and Technology of China, Chengdu, China; ^3^ Department of Pediatric Gastroenterology, Shengjing Hospital of China Medical University, Shenyang, China; ^4^ Department of Gastroenterology, Children’s Hospital of Chongqing Medical University, Chongqing, China; ^5^ Department of Pediatric Gastroenterology, Children’s Hospital of First People’s Hospital of Chenzhou, Chenzhou, China; ^6^ Department of Pediatrics, Zhanjiang Central Hospital, Guangdong Medical University, Zhanjiang, China; ^7^ Department of Gastroenterology, Children’s Hospital of Soochow University, Suzhou, China; ^8^ Department of Pediatrics, Taizhou Hospital of Traditional Chinese Medicine, Taizhou, China; ^9^ Department of Pediatrics, Taihe Hospital, Shiyan, China; ^10^ Department of Gastroenterology, Children’s Hospital of Fudan University, Shanghai, China; ^11^ Department of Gastroenterology, Wuhan Children’s Hospital, Tongji Medical College, Huazhong University of Science and Technology, Wuhan, China; ^12^ Department of Pediatrics, Tongji Hospital of Tongji University, Shanghai, China; ^13^ Department of Pediatrics, First Teaching Hospital of Tianjin University of Traditional Chinese Medicine, Tianjin, China; ^14^ Department of Pediatrics, The Second Affiliated Hospital of Air Force Military Medical University, Xi’an, China; ^15^ Department of Pediatrics, Jinshan Hospital of Fudan University, Shanghai, China; ^16^ Department of Pediatric Gastroenterology, Maternal and Child Health Hospital of Hubei Province, Tongji Medical College, Huazhong University of Science and Technology, Wuhan, China; ^17^ Department of Pediatrics, Shanghai 10th People’s Hospital, Shanghai, China

**Keywords:** Shenqu Xiaoshi oral liquid, traditional Chinese medicine, functional dyspepsia, domperidone syrup, children

Xiao-Li Xie, Jie Wu, Zhong-Yue Li, and Zhi-Gang He were not cited as co-first authors in the published article. The author information has been corrected to denote that Xiao-Li Xie, Jie Wu, Zhong-Yue Li, and Zhi-Gang contributed equally to the article and share first authorship. Additionally, the Author Contributions section has been revised to reflect this amendment. The revised Author Contributions statement appears below.

