# Supplementary material for: Efficacy and Safety of Shenqu Xiaoshi Oral Liquid Compared With Domperidone Syrup in Children With Functional Dyspepsia
Source: Front Pharmacol. 2022 Feb 4;13:831912. doi: 10.3389/fphar.2022.831912 (PMC8854498; doi:10.3389/fphar.2022.831912)
Supplement: Supplementary file 1 [file DataSheet1.docx]

**Supplementary table 1 Assessments schedule**

|  | Screening period | Intervention period | | | Follow-up period |
| --- | --- | --- | --- | --- | --- |
| Visit time | V1 | V2 | V3 | V4 | V5 |
| Visit cycle (day) | -14±2 | Before enrollment (0) | 7 ± 1 | 14 ± 2 | 28 ± 2 |
| Baseline information | × |  |  |  |  |
| Informed consent | × |  |  |  |  |
| Inclusion/exclusion criteria | × | × |  |  |  |
| History/current medical history | × |  |  |  |  |
| Combination medication | × | × | × | × | × |
| Physical examination | × | × | × | × | × |
| Laboratory tests |  | × |  | × |  |
| Electrocardiography |  | × |  | × |  |
| Abdominal ultrasound scan |  | × |  | × |  |
| Clinical evaluation according to the diary card |  | × | × | × | × |
| Adverse event record |  | × | × | × | × |
| Clinical drug dispense |  | × | × |  |  |
| Drug return and assess compliance |  |  | × | × |  |

**Supplementary table 2. The Clinical symptom scoring standard**

| Symptom | severity | | | | frequency | | | |
| --- | --- | --- | --- | --- | --- | --- | --- | --- |
|  | 0 | 1 | 2 | 3 | 0 | 1 | 2 | 3 |
| postprandial fullness | None | Slight fullness | Feeling uncomfortable, but tolerable | unbearable, even unable to bend over | None | 1-2 times a week | 3-5 times a week | ＞5 times a week |
| early satiation | None | Does not affect appetite and food intake | Significantly affect appetite and food intake | severely reduced appetite | None | 1-2 times a week | 3-5 times a week | ＞5 times a week |
| epigastric pain or burning | None | 0<VAS<4 | 4≤VAS<7 | 7≤VAS≤10 | None | 1-2 days a week | 3-5 days a week | ＞5 days a week |
| Loss of appetite | None | poor appetite | aversion to eating | Almost refused to eat |  |  |  |  |
| Reduced food intake (Compared with normal peers) | None | ≥1/3 | 1/2-2/3 | ≥2/3 |  |  |  |  |
| Nausea or vomiting | None | Only nausea | Vomiting several times a week | Vomiting almost every day |  |  |  |  |
| belching |  |  |  |  | None | ＜4 times a day | 4-10 times a day | ＞10 times a day |
| Defecation frequency |  |  |  |  | ≥1 times per day | Once every 2-3 days | Once every 4-6 days | Once more than 7 days |

A 7-day diary is used to assess the severity and frequency of each symptom. The visual analog score (VAS) was used to evaluate pain severity. The VAS comprised 10-cm lines that marked at the extremes no pain and worst pain imaginable. The total weekly score was the sum of the severity score and frequency score of 8 major symptoms, and the severity was scored based on the maximum severity score within a week.

**Supplementary table 3. ANCOVA of individual symptom score in FAS**

|  | ***SXOL group*** | | ***domperidone group*** | | ***Change difference between groups (95% CI)*** | ***P value*** |
| --- | --- | --- | --- | --- | --- | --- |
|  | ***Change from baseline(95% CI)*** | ***P value*** | ***Change from baseline(95% CI)*** | ***P value*** |  |  |
| **Postprandial fullness-severity** |  |  |  |  |  |  |
| **Week 1** | -0.20(-0.28––-0.12) | <0.001 | -0.19(-0.27–-0.11) | <0.001 | -0.00(-0.12–0.11) | 0.966 |
| **Week 2** | -0.36(-0.44–-0.29) | <0.001 | -0.38(-0.46–-0.31) | <0.001 | 0.02(-0.09–0.13) | 0.692 |
| **Week 4** | -0.43(-0.50–-0.35) | <0.001 | -0.46(-0.53–-0.39) | <0.001 | 0.03(-0.07–0.14) | 0.532 |
| **Postprandial fullness-frequency** |  |  |  |  |  |  |
| **Week 1** | -0.18(-0.30–-0.06) | 0.004 | -0.22(-0.34–-0.10) | <0.001 | 0.04(-0.13–0.21) | 0.666 |
| **Week 2** | -0.43(-0.54–-0.33) | <0.001 | -0.45(-0.56–-0.35) | <0.001 | 0.02(-0.13–0.17) | 0.788 |
| **Week 4** | -0.49(-0.59–-0.38) | <0.001 | -0.53(-0.64–-0.43) | <0.001 | 0.05(-0.10–0.20) | 0.534 |
| **Early satiation-severity** |  |  |  |  |  |  |
| **Week 1** | -0.54(-0.63–-0.44) | <0.001 | -0.53(-0.62–-0.43) | <0.001 | -0.01(-0.14–0.13) | 0.895 |
| **Week 2** | -0.70(-0.79–-0.60) | <0.001 | -0.83(-0.93–-0.74) | <0.001 | 0.14(0.00–0.27) | 0.043 |
| **Week 4** | -0.79(-0.88–-0.70) | <0.001 | -0.90(-0.99–-0.81) | <0.001 | 0.11(-0.01–0.24) | 0.079 |
| **Early satiation-frequency** |  |  |  |  |  |  |
| **Week 1** | -0.49(-0.60–-0.39) | <0.001 | -0.57(-0.68–-0.47) | <0.001 | 0.08(-0.07–0.23) | 0.302 |
| **Week 2** | -0.67(-0.78–-0.57) | <0.001 | -0.86(-0.96–-0.75) | <0.001 | 0.19(0.04–0.33) | 0.013 |
| **Week 4** | -0.81(-0.92–-0.70) | <0.001 | -0.87(-0.98–-0.77) | <0.001 | 0.06(-0.09–0.21) | 0.424 |
| **Epigastric pain-severity** |  |  |  |  |  |  |
| **Week 1** | -0.42(-0.50–-0.33) | <0.001 | -0.40(-0.48–-0.32) | <0.001 | -0.02(-0.13–0.10) | 0.773 |
| **Week 2** | -0.61(-0.69–-0.52) | <0.001 | -0.50(-0.58–-0.42) | <0.001 | -0.11(-0.23–0.01) | 0.084 |
| **Week 4** | -0.64(-0.72–-0.57) | <0.001 | -0.67(-0.75–-0.60) | <0.001 | 0.03(-0.08–0.13) | 0.604 |
| **Epigastric pain-frequency** |  |  |  |  |  |  |
| **Week 1** | -0.46(-0.58–-0.35) | <0.001 | -0.49(-0.60–-0.38) | <0.001 | 0.03(-0.13–0.19) | 0.730 |
| **Week 2** | -0.75(-0.85–-0.64) | <0.001 | -0.67(-0.78–-0.57) | <0.001 | -0.07(-0.22–0.07) | 0.331 |
| **Week 4** | -0.83(-0.92–-0.74) | <0.001 | -0.83(-0.92–-0.74) | <0.001 | -0.00(-0.13–0.13) | 0.998 |
| **Loss of appetite-severity** |  |  |  |  |  |  |
| **Week 1** | -0.45(-0.53–-0.36) | <0.001 | -0.45(-0.53–-0.37) | <0.001 | 0.01(-0.11–0.12) | 0.892 |
| **Week 2** | -0.62(-0.69–-0.54) | <0.001 | -0.72(-0.80–-0.65) | <0.001 | 0.11(-0.00–0.21) | 0.051 |
| **Week 4** | -0.71(-0.78–-0.64) | <0.001 | -0.79(-0.86–-0.72) | <0.001 | 0.09(-0.01–0.18) | 0.085 |
| **Reduced food intake-severity** |  |  |  |  |  |  |
| **Week 1** | -0.60(-0.69–-0.51) | <0.001 | -0.55(-0.64–-0.46) | <0.001 | -0.05(-0.18–0.08) | 0.453 |
| **Week 2** | -0.87(-0.96–-0.79) | <0.001 | -0.84(-0.92–-0.76) | <0.001 | -0.04(-0.15–0.08) | 0.528 |
| **Week 4** | -0.93(-1.01–-0.85) | <0.001 | -0.92(-0.99–-0.84) | <0.001 | -0.01(-0.12–0.10) | 0.810 |
| **Nausea or vomiting-severity** |  |  |  |  |  |  |
| **Week 1** | -0.19(-0.26–-0.12) | <0.001 | -0.21(-0.28–-0.14) | <0.001 | 0.02(-0.07–0.12) | 0.675 |
| **Week 2** | -0.30(-0.36–-0.25) | <0.001 | -0.23(-0.29–-0.18) | <0.001 | -0.07(-0.15–0.01) | 0.075 |
| **Week 4** | -0.31(-0.35–-0.26) | <0.001 | -0.32(-0.37–-0.28) | <0.001 | 0.02(-0.05–0.08) | 0.630 |
| **Belching-frequency** |  |  |  |  |  |  |
| **Week 1** | -0.12(-0.19–-0.04) | 0.003 | -0.10(-0.17–-0.03) | 0.009 | -0.02(-0.12–0.09) | 0.763 |
| **Week 2** | -0.24(-0.30–-0.18) | <0.001 | -0.16(-0.21–-0.10) | <0.001 | -0.08(-0.16–0.00) | 0.062 |
| **Week 4** | -0.24(-0.30–-0.18) | <0.001 | -0.21(-0.27–-0.15) | <0.001 | -0.03(-0.11–0.06) | 0.518 |
| **Defecation frequency** |  |  |  |  |  |  |
| **Week 1** | -0.08(-0.14–-0.01) | 0.022 | -0.10(-0.16–-0.03) | 0.003 | 0.02(-0.07–0.11) | 0.617 |
| **Week 2** | -0.16(-0.23–-0.09) | <0.001 | -0.13(-0.20–-0.06) | <0.001 | -0.03(-0.13–0.07) | 0.569 |
| **Week 4** | -0.16(-0.23–-0.09) | <0.001 | -0.12(-0.18–-0.05) | 0.001 | -0.05(-0.15–0.05) | 0.356 |
